# Molecular epidemiology of coxsackievirus A16 circulating in children in Beijing, China from 2010 to 2019

**DOI:** 10.1007/s12519-021-00451-y

**Published:** 2021-08-28

**Authors:** Ya-Fang Hu, Li-Ping Jia, Fang-Yuan Yu, Li-Ying Liu, Qin-Wei Song, Hui-Jin Dong, Jie Deng, Yuan Qian, Lin-Qing Zhao, Li Deng, Hui Huang, Ru-Nan Zhu

**Affiliations:** 1 Laboratory of Virology, Beijing Key Laboratory of Etiology of Viral Diseases in Children, Capital Institute of Pediatrics, 2 Yabao Road, Chaoyang District, Beijing 100020, China; 2 Department of Clinical Laboratory, Shanghai Children’s Hospital, Shanghai Jiaotong University, Luding Road 355, Putuo District, Shanghai 200040, China; 3 Department of Clinical Laboratory, Children’s Hospital of Capital Institute of Pediatrics, 2 Yabao Road, Beijing 100020, China; 4 Department of Infectious Diseases, Children’s Hospital of Capital Institute of Pediatrics, 2 Yabao Road, Beijing 100020, China

**Keywords:** Coxsackievirus A16, Genetic evolution, Molecular epidemiology, Phylogenetic analysis

## Abstract

**Background:**

Coxsackievirus A16 (CVA16) is one of the major etiological agents of hand, foot and mouth disease (HFMD). This study aimed to investigate the molecular epidemiology and evolutionary characteristics of CVA16.

**Methods:**

Throat swabs were collected from children with HFMD and suspected HFMD during 2010–2019. Enteroviruses (EVs) were detected and typed by real-time reverse transcription-polymerase chain reaction (RT-PCR) and RT-PCR. The genotype, evolutionary rate, the most recent common ancestor, population dynamics and selection pressure of CVA16 were analyzed based on viral protein gene (*VP1*) by bioinformatics software.

**Results:**

A total of 4709 throat swabs were screened. EVs were detected in 3180 samples and 814 were CVA16 positive. More than 81% of CVA16-positive children were under 5 years old. The prevalence of CVA16 showed obvious periodic fluctuations with a high level during 2010–2012 followed by an apparent decline during 2013–2017. However, the activities of CVA16 increased gradually during 2018–2019. All the Beijing CVA16 strains belonged to sub-genotype B1, and B1b was the dominant strain. One B1c strain was detected in Beijing for the first time in 2016. The estimated mean evolutionary rate of *VP1* gene was 4.49 × 10^–3^ substitution/site/year. Methionine gradually fixed at site-23 of VP1 since 2012. Two sites were detected under episodic positive selection, one of which (site-223) located in neutralizing linear epitope PEP71.

**Conclusions:**

The dominant strains of CVA16 belonged to clade B1b and evolved in a fast evolutionary rate during 2010–2019 in Beijing. To provide more favorable data for HFMD prevention and control, it is necessary to keep attention on molecular epidemiological and evolutionary characteristics of CVA16.

**Supplementary Information:**

The online version contains supplementary material available at 10.1007/s12519-021-00451-y.

## Introduction

Enterovirus (EV) is a common pathogen, and is responsible for many infectious diseases in human, including hand, foot and mouth disease (HFMD), herpangina (HA), acute hemorrhagic conjunctivitis, respiratory infections, acute myocarditis, meningitis, encephalitis and acute flaccid paralysis [1]. HFMD was classified as a statutorily notifiable infectious disease in China in 2008 [2]. The clinical manifestations of HFMD are highly complex and heterogeneous, which makes it difficult for doctors to give an exact clinical diagnosis, especially at the early stage of disease. The main manifestations of HFMD are fever and rash on hands, feet, mouth, and buttocks, however, central nervous system complications and cardiopulmonary failure may occur in severe cases [3]. Therefore, early recognition of cases and identification of the pathogens are the key to prevention and control for HFMD.

Coxsackievirus A16 (CVA16) is a member of species *Enterovirus A* in genus *Enterovirus*, family *Picornaviridae*. As one of the main pathogens of HFMD, CVA16 was responsible for several HFMD outbreaks in the world, especially in Asia–Pacific region [4–6]. HFMD caused by CVA16 infection is generally mild and self-limiting. However, CVA16 can occasionally cause severe and fatal cases [7]. CVA16 contains a positive sense, single-stranded RNA genome [8]. The nucleotide sequence of viral protein gene (*VP1*), encoding the most important structural protein, is well correlated with EV serotype and genotype [9, 10]. Up to now, global CVA16 strains can be divided into three genotypes, A, B and D based on the phylogenetic tree and genetic diversity of *VP1* gene. Genotype B can be further divided into three sub-genotypes, B1, B2 and B3. Sub-genotype B2 was the predominant type before 2000, then replaced by B1 strains [11]. Sub-genotype B1 contains clade B1a, B1b and B1c. Two novel CVA16 strains isolated by Chen et al. in 2017 in Shenzhen, China, were designated as sub-genotype B3 by the phylogenetic reconstruction of *VP1* [12]. Genotype D was first detected in Peru in 2009, then circulated in France from 2011 to 2014 [13]. In 2016, the first outbreak and spread of genotype D in China was reported in Shanghai [14].

Our laboratory has been conducting etiological surveillance for HFMD and EV-associated infectious diseases since 2007 [15, 16]. During the monitoring period, more types of EVs apart from EV-A71 and CVA16 were further identified along with the continuous modification of primers and polymerase chain reaction (PCR) amplification conditions [17]. This study focused on the molecular epidemiology and evolutionary features of CVA16 circulating in children with HFMD and suspected HFMD in Beijing from 2010 to 2019.

## Methods

### Patients selection and samples collection

Patients involved in this study visited the Department of Infectious Diseases, Children's Hospital of Capital Institute of Pediatrics during the period from March 2010 to October 2019. Throat swabs were collected from patients who were under 18 years old with clinical diagnoses of HFMD, HA and rash and fever illness. Among 4709 patients, the ratio of male and female was 1.39:1. The mean age was 3.35 years (range from 9 days to 17 years 6 months).

### Enterovirus detection and typing

The processes for EV detection and typing have been described previously [17]. Briefly, viral RNA was extracted from clinical samples using QIAamp Viral RNA Mini Kit (QIAGEN, Germany), and real-time reverse transcription (RT)-PCRs for pan-EV, EV-A71, CVA16, CVA6 and CVA10 were performed. Samples, which were only positive for pan-EV, were further typed using RT-PCR and sequencing.

### *VP1* gene amplification and sequencing

CVA16-positive samples were selected randomly and proportionally each year to amplify the complete *VP1* gene using primers CVA16-VP1-F and CVA16-VP1-R (or CVA16-VP1-F1 and CVA16-VP1-R1) (Table 1). The positive PCR products were sequenced by Sino Geno Max Co. Ltd. (Beijing, China). The *VP1* sequences (891 bp) were edited using DNAStar v. 5.01 software and compared with publicly available sequences in GenBank using BLAST (http://www.ncbi.nlm.nih.gov/BLAST/). The similarity and divergence of nucleotide and deduced amino acid sequences were estimated by MEGA v. 6.05 software (*P*-distance model) [18].

**Table 1:** Primers used for CVA16 *VP1* gene amplification reactions

Primers	Nucleotide sequence (5'→3')	Length (bp)
CVA16-VP1-F	CYATGAAACTRTGCAAGG	1014
CVA16-VP1-R	TGGCAAGGTGYCGATTCA	
CVA16-VP1-F1^a^	TATGTNGTRCCYATTGGTG	1075
CVA16-VP1-R1^a^	GTCGRTTCACYACCCTRTA	

*CVA16* coxsackievirus A16, *VP* viral protein. ^a^CVA16-VP1-F1 and CVA16-VP1-R1 were optimized primers

### Phylogenetic analysis

Phylogenetic tree was constructed by neighbor-joining (NJ) and maximum likelihood (ML) methods based on Kimura 2-parameter model using MEGA v. 6.05 software. The bootstrap analyses with 1000 repetitions were performed to estimate the reliability of the phylogenetic inference at each branch node.

### Molecular evolution and population dynamics analysis

To assess whether there was sufficient temporal signal in the *VP1* sequence dataset to proceed with molecular clock analysis, a regression analysis of root-to-tip genetic distance against sampling date based on the ML tree of Beijing strains was performed using TempEst v. 1.5.3 software [19]. The Bayesian molecular clock phylogeny and evolutionary rate of CVA16 *VP1* genes were analyzed using Markov chain Monte Carlo method in BEAST v. 1.10.4 software [20]. The chain length was 100 million steps with sampling every 10,000 steps. The substitution model was selected using jModelTest v. 2.1.10 software based on the Akaike information criterion and Bayesian information criteria values [21]. As a result, the datasets were analyzed using Hasegawa-Kishino-Yano + G + I substitution model under a lognormal distributed uncorrelated relaxed clock model. Tracer v. 1.7.1 program was used to check for convergence. Effective sample size > 200 for all inferred parameters was accepted. Maximum clade credibility (MCC) tree was calculated with Tree Annotator v. 1.10.4 after the removal 10% of sample burn-in. To estimate the effective population size (EPS) of CVA16 circulating in Beijing, a Bayesian skyline plot (BSP) was reconstructed using Tracer v. 1.7.1 program.

### Selection pressure analysis

Natural selected sites were inferred by mixed-effects model for episodic diversifying selection (MEME), single likelihood ancestor counting (SLAC) and fast unconstrained Bayesian approximation (FUBAR) methods implemented in Datamonkey online server (http://datamonkey.org/) [22–24]. MEME employs a mixed-effects maximum likelihood approach which is capable of identifying both episodic and pervasive selection at the level of an individual site. SLAC uses a combination of maximum likelihood and counting approaches with the most rigorous test result. FUBAR uses a Bayesian approach which can avoid misleading inference due to model misspecification. Both SLAC and FUBAR assume that the selection pressure for each site is constant along the entire phylogeny, estimating pervasive selection at the level of an individual site. Positively selected sites were determined by a *P* value of < 0.05 (MEME and SLAC methods) or a posterior probability of > 0.9 (FUBAR method).

## Results

### Epidemiology

**Fig. 1:**
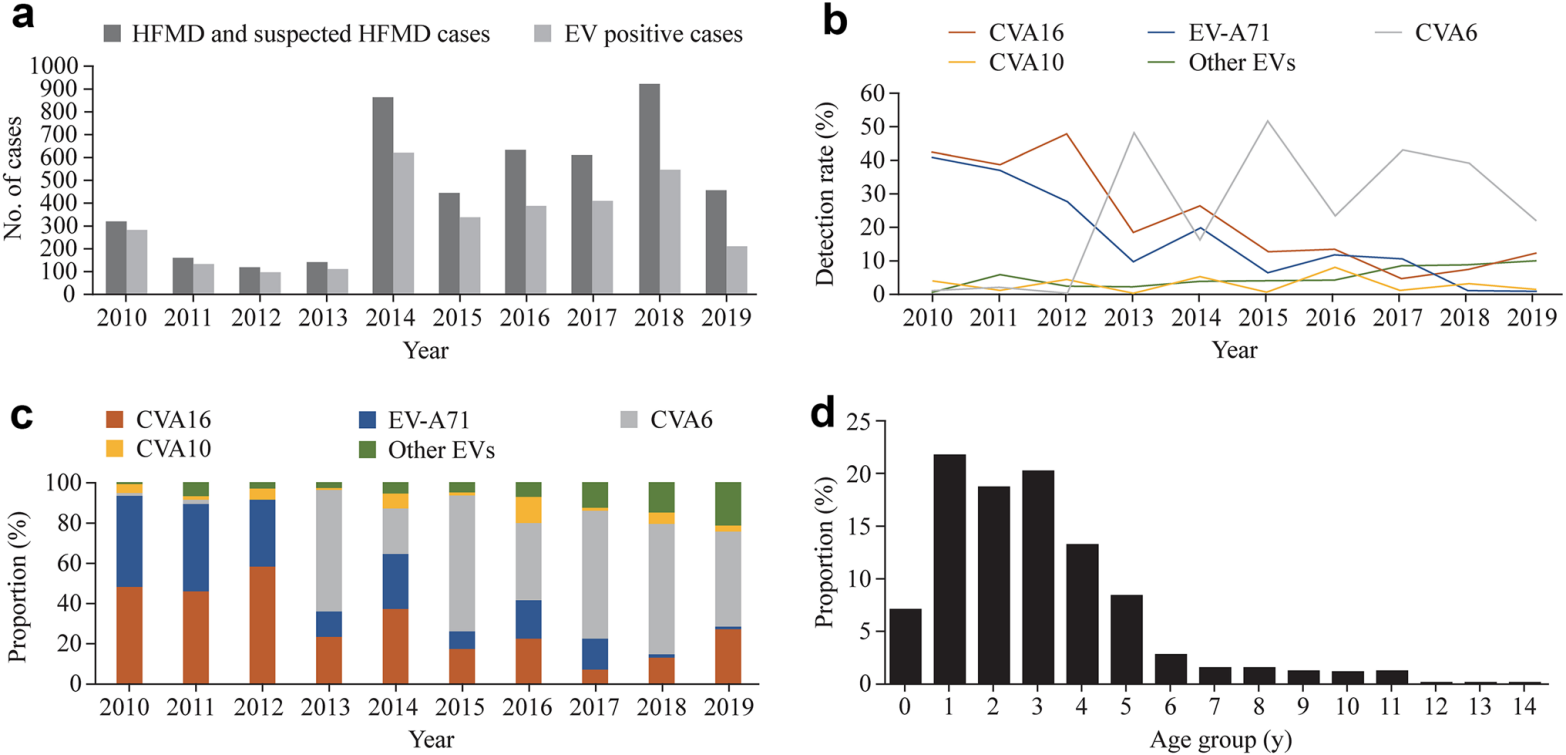
Epidemiological features of EVs circulating in Beijing children with HFMD and suspected HFMD during 2010–2019. **a** Number of screening cases and positive cases for EVs; **b** the detection rate of EVs (including CVA16, CVA6, CVA10, EV-A71 and other EVs); **c** proportion of CVA16, CVA6, CVA10, EV-A71 and other EVs in EV-positive patients; **d** the age distribution of CVA16-positive children. *EV* enteroviruses, *HFMD* hand, foot and mouth disease, *CVA* coxsackievirus

A total of 4709 throat swabs were collected from children with clinical diagnoses of HFMD, HA and rash and fever illness from March 2010 to October 2019. EVs were detected in 67.5% (*n* = 3180) of samples, and 17.3% (*n* = 814) were positive for CVA16. Figure 1a shows the annual number of collected cases and EV-positive cases. The number of screening and laboratory confirmed EV-positive cases after 2013 significantly increased because of the HFMD outbreak of CVA6 in China and the atypical clinical manifestation caused by CVA6 [25, 26]. During 2010–2012, the detection rates of CVA16 were at high level (42.4%, 39.0%, 47.9%, respectively), while the detection rate of CVA6 was extremely low. In 2013, the detection rate of CVA16 dropped rapidly (18.4%), along with a sharp rise of CVA6 (48.2%) (Fig. 1b). In 2014, the detection rate of CVA16 increased again, and that of CVA6 dropped rapidly. With a fluctuating decline, the detection rate of CVA16 decreased to the lowest level in 2017 (4.6%), followed by an increase to 12.6% in 2019, which was higher than the detection rate in 2018 (7.5%). Figure 1c shows the change of the proportion of CVA16, CVA6, EV-A71, CVA10 and other EVs over time. From 2010 to 2011, CVA16 and EV-A71 were both the main pathogens with similar proportion, then CVA16 became the predominant pathogen in 2012. Since 2013, CVA6 became the main pathogen replacing CVA16 and EV-A71 (except in 2014). Although increasing slightly in 2014, the proportion of CVA16 continually maintained at low level during 2015–2019. It is worth noting that the proportion of CVA16 rose gradually during 2018–2019, which was in accordance with the detection rate.

Among the CVA16-positive children, 789 cases with complete demographic information were selected to analyze age and sex distributions. Male to female ratio was 1.28:1. The median age was 3.0 years old (range from 1 month to 14 years 2 days). 81.6% (644/789) were younger than 5 years old. Most of them were in the age of 1–3 years old. The proportion of CVA16 infection declined with age among children older than 4 years old (Fig. 1d).

### Phylogenetic analysis of *VP1* sequence

Totally, 172 *VP1* nucleotide sequences were acquired, including 20 (14.7% of 136) collected in 2010, 13 (21.0% of 62) in 2011, 12 (21.1% of 57) in 2012, 13 (50.0% of 26) in 2013, 26 (11.2% of 232) in 2014, 10 (17.2% of 58) in 2015, 19 (21.8% of 87) in 2016, 9 (32.1% of 28) in 2017, 26 (37.1% of 70) in 2018 and 24 (41.4% of 58) in 2019. The full length of *VP1* gene was 891 bp without insertion or deletion. Sequence similarities were 87.0–100% at nucleotide level and 97.6–100% at amino acid level. Eight pairs of sequences shared the same nucleotide sequence, respectively. The 172 *VP1* sequences were deposited in GenBank database under accession numbers MT553115–MT553241, MN886521–MN886522, MW462132–MW462168.

Phylogenetic tree was constructed using NJ method based on the alignment of 164 unduplicated *VP1* sequences generated in this study. A total of 95 reference sequences were obtained from GenBank database including the *VP1* sequences of prototype G-10 strain, global CVA16 strains and the prototype strain of EV-A71 (BrCr). Five additional sequences collected in Beijing between 2010 and 2014 (obtained from GenBank database) were also included. The NJ tree in Supplementary Fig. 1 shows that all the 169 Beijing CVA16 strains belonged to B1 sub-genotype, and attached to 3 clades (B1a, B1b and B1c). Overall, 16 (9.0%) out of 177 Beijing strains (including the 8 duplicate sequences described above) belonged to clade B1a, limitedly circulating in 2010, 2011, 2013, 2017 and 2019 in Beijing. Only one B1c strain (S3540) was detected in 2016, closest to an Indian strain collected in 2015, suggesting a possible source of transmission. The remaining strains belonged to B1b, as the predominant clade circulating during 2010–2019 (Table 2).

**Table 2:** Distribution and proportion of the clades of sub-genotype B1 of CVA16 circulating in Beijing during 2010–2019

Year	Number of strains			Proportion		
	B1a	B1b	B1c	B1a	B1b	B1c
2010	6	15	–	28.57	71.43	–
2011	5	11	–	31.25	68.75	–
2012	–	12	–	–	100.00	–
2013	2	11	–	15.38	84.62	–
2014	–	27	–	–	100.00	–
2015	–	10	–	–	100.00	–
2016	–	18	1	–	94.74	5.26
2017	1	8	–	11.11	88.89	–
2018	–	26	–	–	100.00	–
2019	2	22	–	8.33	91.67	–
Total	16	160	1	9.04	90.40	0.56

*CVA16* coxsackievirus A16. “-” no strain was found

Nucleotide and deduced amino acid divergences of *VP1* were described in Table 3. All of the Beijing and reference strains including the prototype EV-A71 strain (BrCr) were analyzed. As a result, the mean nucleotide divergences between genotypes A, B and D were higher than 14.0%, the mean nucleotide divergences between sub-genotypes B1, B2 and B3 were greater than or equal to 10.0%, and less than 10.0% of the mean nucleotide divergence was existed between clades B1a, B1b and B1c. The divergences of the deduced VP1 amino acid sequences between genotypes A and B, A and D were obvious; however, the divergence between genotypes B and D was small, similar to that of intra-genotype B, indicating the closer evolutionary relationship between genotypes B and D.

**Table 3:** The divergence of *VP1* among different genotypes of CVA16 and between CVA16 and EV-A71

Variables	Nucleotide divergence (%)	Amino acid divergence (%)
Inter-serotype divergence		
EV-A71 (BrCr)-CVA16^a^	34.3 (33.0–38.4)	29.0 (28.3–29.7)
Inter-genotype divergence of CVA16^a^		
A–B	24.2 (22.2–25.9)	8.7 (7.1–10.5)
A–D	25.2 (24.7–25.8)	8.6 (7.7–9.4)
B–D	14.1 (12.1–17.3)	0.9 (0.3–2.4)
Inter-sub-genotype divergence of CVA16^a^		
B1–B2	10.9 (5.8–15.9)	0.8 (0–2.4)
B1–B3	10.0 (4.6–12.6)	1.0 (0–2.7)
B2–B3	11.0 (8.6–12.8)	1.0 (0–2.0)
Inter-clade divergence of CVA16^a^		
B1a–B1b	8.6 (3.1–12.9)	0.7 (0–2.4)
B1a–B1c	9.5 (5.3–12.3)	1.4 (0.7–2.7)
B1b–B1c	9.3 (5.4–12.2)	1.6 (0.7–3.0)

Values are median (range). *VP* viral protein, *CVA16* coxsackievirus A16, *EV-A71* enterovirus A71. ^a^The Beijing and reference CVA16 strains in this study

The consensus amino acid variations of VP1 in different branches were shown on the right in Supplementary Fig. 1. The amino acid at site-13 was different in the strains among different genotypes A, B and D. At site-14, a separate cluster of B1b strains had a consistent N14S mutation, becoming the same amino acid as genotype A strains. A wider cluster of B1b strains contained a consistent mutation (L23M) at site-23. The amino acid at site-23 of the B1b strains collected in Beijing from 2010 to 2011 was mainly leucine (L), whereas most of the strains emerged L23M mutation in 2012 (Table 4). Methionine (M) at site-23 was fixed in the majority of the B1b strains collected from 2013 to 2019. All strains with the amino acid mutation mentioned above belonged to cluster 3 in Supplementary Fig. 2a. Compared with other strains, the B1c strains had synchronous mutations of I235V and T240A.

**Table 4:** Proportion of CVA16 B1b strains with the mutation at site-23 on VP1 protein in Beijing

Year	Site-23	
	Leucine, %	Methionine, %
2010	100	0
2011	82	18
2012	33	67
2013	9	82
2014	0	100
2015	0	100
2016	0	100
2017	0	100
2018	4	92
2019	0	95

*VP* viral protein, *CVA16* coxsackievirus A16

### Molecular evolution and population dynamics

For the 169 Beijing CVA16 strains collected during 2010–2019, a Bayesian MCC tree based on *VP1* gene was generated to confirm the evolutionary relationship and to explore the timescale of CVA16. The correlation coefficient calculated by TempEst was 0.899, and the root-to-tip plot showed a positive correlation between genetic divergence and sampling time (Supplementary Fig. 3), suggesting that the dataset was suitable for phylogenetic molecular clock analysis. As shown in Supplementary Fig. 2a, the time-scaled MCC tree contains three clades corresponding to clades B1a, B1b and B1c in Supplementary Fig. 1. Clade B1b can be further divided into 3 clusters (cluster 1, cluster 2 and cluster 3). The strains that emerged earlier (2010–2013) are mostly located at the root of the MCC tree. Most strains (cluster 3) that emerged later evolved from early strains, and further formed multiple new branches. Cluster 3 of clade B1b became the predominant strains since 2014, and formed a sustained epidemic through multiple transmission chains in the local area. However, there was no obvious ladder-like topological structure for clade B1a on MCC tree, indicating that B1a strains had a limited prevalence in Beijing, most of which may be imported from the other regions.

Based on the Bayesian coalescent approach, we assessed the evolutionary features of the CVA16 circulating in Beijing. The most recent common ancestor of CVA16 emerged approximately in 1996 [95% highest posterior density (HPD): 1992–2000]. The mean evolutionary rate was estimated to be 4.49 × 10^–3^ substitution/site/year (95% HPD: 3.98 × 10^–3^–4.98 × 10^–3^).

The BSP based on the *VP1* gene shows the change in genetic diversity of *VP1* gene and EPS of CVA16, reflecting the dynamic change of the viral population circulating in Beijing during 2010–2019 (Supplementary Fig. 2b). During the ten-year period, the EPS of CVA16 experienced two obvious fluctuations. The EPS of CVA16 kept at a high level during 2010–2012 and dropped to the lowest point in 2013. A rapid rise appeared in 2014 and kept at a high level during 2014–2016, followed by an obvious decrease in 2017. From 2018 to 2019, the EPS presented a slight and slow rise. Compared the epidemiological data (Fig. 1b) with BSP, the fluctuation trend of EPS basically coincided with that of the detection rate of CVA16.

### Selection pressure analysis of CVA16 strains in Beijing

Over the past decade, CVA16 continuously circulated in Beijing. To understand the natural selection pressure on *VP1* gene, 169 unduplicated *VP1* nucleotide sequences of Beijing CVA16 strains described above were analyzed by Datamonkey. The results of SLAC and FUBAR analyses showed no positive selection site on VP1 and most sites were under negative selection (204 and 275 sites, respectively). The result of SLAC analysis showed that the overall dN/dS ratio of *VP1* gene is very low (0.02). These results above suggest that *VP1* gene of Beijing CVA16 strains was subjected to very strong purifying selection. However, two sites under episodic positive selection were detected by MEME. The positively selected amino acid substitutions were D2V and G223N distributed in two strains (S854 sampled in 2010 and S5822 sampled in 2018) (marked in Supplementary Fig. 1). The mutation at site-223 was located on the linear neutralizing epitope on the surface of the virus capsid (PEP71, aa. 211–225 [27]), resulting in the change of non-polar hydrophobic amino acid (G) to polar neutral amino acid (N).

## Discussion

This retrospective study investigated the molecular epidemiology and genetic evolutionary characteristics of CVA16 circulating in children diagnosed with HFMD and suspected cases in Beijing during 2010–2019. Over the past decade, the prevalence of CVA16 has changed significantly. CVA16 and EV-A71 were replaced by CVA6 in 2013 and during 2015–2019. On the whole, CVA16 had a fluctuating prevalence pattern in Beijing from 2010 to 2018, with the detection rate increasing every other year. The above prevalence pattern also existed in the other regions of China [28, 29]. CVA6 and CVA10 also had the same trend, but the detection rate of CVA10 was always at a low level. However, different countries may have different epidemic patterns due to the differences in climate and medical-health conditions [30]. The detection rate of EV-A71 dropped obviously during 2017–2019, in line with another research about the application of EV-A71 vaccine in China [31]. However, the prevalence of CVA16 showed an upward trend from 2018 to 2019. It suggests that EV-A71 vaccine has limited cross-protection effect on CVA16. Therefore, it is necessary to use a multivalent vaccine that can prevent common types of EVs. Most children with CVA16 infection aged younger than 5 years, and younger children (1–3 years old) accounted for a larger proportion, consistent with HFMD-related studies in China and other Asian countries [32, 33].

The global CVA16 strains can be divided into 3 genotypes, and genotype B can be divided into 3 sub-genotypes. In recent years, sub-genotype B1 was the prevalent strain worldwide [4], also contributed to all the CVA16 infections in this study. Clade B1a was first reported in Japan in 1995, then became the dominant strain in mainland China, Malaysia and Thailand [34–36]. B1b, the complex recombinant of CVA16 with CVA4 and EV-A71, co-circulated with B1a in China during 1999–2008 [37, 38]. After 2010, B1b became the predominant strain in China [12, 39, 40]. Clade B1c strain was first emerged in Malaysia (2005–2007), then detected in several countries, such as Russia, France, Indian and Japan, and induced an outbreak of HFMD in Indian in 2013 [34, 41, 42]. Recently, a B1c strain sampled in 2017 was reported in the west area of China [29]. In this study, clade B1b was the dominant strain during 2010–2019, circulated in Beijing and evolved continuously, while the prevalence of clade B1a and B1c strains were limited. Clade B1c and genotype D of CVA16 were newly emerged types in recent years, and transmitted in several countries [13, 14, 42]. Genotype D strains experienced multiple recombination during transmission, which might change the biological characteristics of the virus, then change the spreading ability and pathogenicity of the virus [43]. Therefore, it should be paid close attention to the molecular epidemiology of CVA16 to timely detect and control the potential HFMD outbreak caused by the new genotypes of CVA16.

The divergence of *VP1* nucleotide sequences greater than 15% is one of the reference basis for CVA16 genotyping [10, 38]. However, in this research, the divergence between strains of genotype B and D is slightly lower than 15%, and the divergence of amino acid sequences was relatively low. It might be due to the recombinant origin of genotype D [13]. The considerable overlap between the nucleotide sequence divergence of sub-genotypes (B1–B3) and clades (B1a–B1c) suggested that it was difficult to distinguish sub-genotype and clade only by the divergence of *VP1* nucleotide sequences.

In this study, the emerging time of CVA16 strain in Beijing was about 1996 (95% HPD: 1992–2000), which was similar to the report in terms of mainland China (1994, 95% HPD: 1986–1999) and the emerging time of sub-genotype B1 based on the worldwide data (1992, 95% HPD: 1990–1994) [13, 29]. EVs are among the most rapidly evolving viruses, as the evolutionary rate of partial *VP1* gene was in the range of 4.1 × 10^–3^–12.2 × 10^–3^ substitutions/site/year [44]. The evolutionary rate of *VP1* gene (genotype B) in the mainland of China (sampled during 2000–2018) was 3.74 × 10^–3^ substitutions/site/year [29]. Zhao et al. used worldwide data (sampled during 1951–2013) for analysis and found the mean evolutionary rate of CVA16 was 6.656 × 10^–3^ substitutions/site/year [41]. In this study, the mean evolutionary rate of CVA16 circulating in Beijing from 2010 to 2019 was 4.49 × 10^–3^ substitutions/site/year, which is similar to that in Yunnan Province, China from 2009 to 2015 (4.545 × 10^–3^ substitutions/site/year) and France from 2010 to 2014 (4.5 × 10^–3^ substitutions/site/year) [13, 39].

The BSP based on *VP1* gene of CVA16 can reflect the pathogen population dynamics. The BSP reconstructed using CVA16 *VP1* gene worldwide sampled from 1980 to 2013 showed the relationship between the viral genetic diversity and the outbreak of HFMD caused by CVA16. Every sharp increase of CVA16 genetic diversity reflected the emergence of a new genotype, which resulted in a large-scale HFMD outbreak [41]. In mainland China, CVA16 genetic diversity increased from 2000 to 2009, then decreased gradually from 2010 to 2013. From 2014 to 2016, it continued to increase again, then decreased slightly from 2017 to 2018 [29]. The genetic diversity correlated with EPS. There was a similar result in this study. The EPS was at a relatively high level from 2014 to 2016, but the detection rate of CVA16 did not reach a correspondingly high level. It may be due to the increased focus on atypical HFMD cases caused by CVA6 and increased efforts to screening for EVs after 2013. It should be noted that the EPS and prevalence trend of CVA16 continued to increase from 2018 to 2019 in Beijing, therefore, implying the enhancement of CVA16 activity and the raising alertness about the potential outbreak.

Site-23 of VP1 is located at the N-terminus of the protein, which is related to the stabilization of the capsid structure and the process of RNA release [45, 46]. The prevalence of CVA16 in Beijing was related to the spread of cluster 3 of clade B1b from 2014 to 2019, all of which had L23M mutation. This mutation was also found in an outbreak of CVA16 in Taizhou from 2011 to 2013 [47]. Whether it is beneficial for virus transmission remains to be analyzed in depth.

VP1 protein of EV contains major epitopes and is mainly affected by the selective pressure of the host's immune system. Most mutations were not beneficial for immune escape of the virus. One site under episodic positive selection pressure (site-223) was located in the linear neutralizing epitope PEP71 [27]. Whether this mutation (G223N) emerged in 2018 could increase the chance of immune escape of the virus and continue to accumulate in the virus population requires close monitoring. The site subject to positive selection may affect the protective effect of the vaccine [48]. Therefore, it is necessary to find suitable neutralizing epitopes to develop effective subunit vaccine, nucleic acid vaccine, etc. Other neutralizing epitopes (PEP32, PEP37, PEP55, PEP63 and PEP91) of VP1 protein, under purifying selection pressure, were more suitable as candidate epitopes for vaccine design than PEP71.

This study had two limitations. First, the detailed clinical information of CVA16-positive patients was incomplete, which limited the further analysis of the clinical symptoms. Second, only local CVA16 strains were involved in this study to analyze the evolutionary characteristics, which limited to understand the transmission and evolution worldwide.

In conclusion, this study summarized the molecular epidemiological and genetic evolutionary characteristics of Beijing CVA16 strains circulating between March 2010 and October 2019. Although CVA16 is not the predominant pathogen of HFMD in recent years, there could be an outbreak of CVA16 in the future due to the lack of available vaccine, accumulation of the susceptible host population, continuous evolution of the virus and import of new genotypes. Therefore, it is necessary to keep attention on molecular epidemiological and evolutionary characteristics of CVA16.

## Supplementary Information

Below is the link to the electronic supplementary material.

**Supplementary Fig. 1 **Neighbor-joining (NJ) phylogenetic tree based on the nucleotide sequences of the CVA16 *VP1* gene and alignment of the deduced amino acid residues of VP1 protein. Duplicate sequences obtained in this study were deleted. The prototype enteroviruses A71 strain (BrCr) was used as an outgroup. The nucleotide substitution model was Kimura 2-parameter. A bootstrapping analysis was performed 1000 pseudo-replicated datasets. Only bootstrap support values ≥ 70% were shown. The strains collected in Beijing during 2010–2019 were marked with red circle. Deduced VP1 amino acid residues alignment was listed on the right. Mutations that are specific among genotypes/sub-genotypes and under positive selection pressure are highlighted with green. *CVA16* coxsackievirus A16, *VP* viral protein (TIF 13940 kb)

**Supplementary Fig. 2 **Genetic evolutionary analyses of the *VP1* gene of CVA16 circulating in Beijing children during 2010–2019. **a** MCC tree based on Bayesian Markov chain Monte Carlo analysis;** b **Bayesian skyline plot showing the EPS of CVA16. The deep blue line is the median value of the estimated EPS. The light blue shade represents the upper and the lower estimates of 95% intervals. *VP* viral protein, *CVA16* coxsackievirus A16, *MCC* maximum clade credibility, *EPS* effective population size (TIF 7967 kb)

**Supplementary Fig. 3 **Root-to-tip regression analysis for the temporal signal of the *VP1* gene of CVA16 circulating in Beijing children during 2010-2019 calculated by TempEst. *VP* viral protein, *CVA16* coxsackievirus A16 (TIF 670 kb)

## References

[1] Pallansch MA, Oberste MS, Whitton JL, Knipe DM, Howley PM (2013). Enteroviruses: polioviruses, coxsackieviruses, echoviruses, and newer enteroviruses. Fields virology.

[2] Xing W, Liao Q, Viboud C, Zhang J, Sun J, Wu JT (2014). Hand, foot, and mouth disease in China, 2008–12: an epidemiological study. Lancet Infect Dis.

[3] Li XW, Ni X, Qian SY, Wang Q, Jiang RM, Xu WB, et al. Chinese guidelines for the diagnosis and treatment of hand, foot and mouth disease (2018 edition). World J Pediatr. 2018;14:437–47. 10.1007/s12519-018-0189-8 30280313

[4] Fu X, Wan Z, Li Y, Hu Y, Jin X, Zhang C (2020). National epidemiology and evolutionary history of four hand, foot and mouth disease-related enteroviruses in China from 2008 to 2016. Virol Sin.

[5] Van Tu P, Thao NTT, Perera D, Truong KH, Tien NTK, Thuong TC (2007). Epidemiologic and virologic investigation of hand, foot, and mouth disease, southern Vietnam, 2005. Emerg Infect Dis.

[6] Cabrerizo M, Tarragó D, Muñoz-Almagro C, Del Amo E, Domínguez-Gil M, Eiros JM (2014). Molecular epidemiology of enterovirus 71, coxsackievirus A16 and A6 associated with hand, foot and mouth disease in Spain. Clin Microbiol Infect.

[7] Mao Q, Wang Y, Yao X, Bian L, Wu X, Xu M (2014). Coxsackievirus A16: epidemiology, diagnosis, and vaccine. Hum Vaccin Immunother.

[8] Kitamura N, Semler BL, Rothberg PG, Larsen GR, Adler CJ, Dorner AJ (1981). Primary structure, gene organization and polypeptide expression of poliovirus RNA. Nature.

[9] Oberste MS, Maher K, Kilpatrick DR, Pallansch MA (1999). Molecular evolution of the human enteroviruses: correlation of serotype with VP1 sequence and application to picornavirus classification. J Virol.

[10] Perera D, Yusof MA, Podin Y, Ooi MH, Thao NT, Wong KK (2007). Molecular phylogeny of modern coxsackievirus A16. Arch Virol.

[11] Sun T, Liu Y, Zhang Y, Zhou L (2014). Molecular phylogeny of coxsackievirus A16. J Clin Microbiol.

[12] Chen L, Yao XJ, Xu SJ, Yang H, Wu CL, Lu J (2019). Molecular surveillance of coxsackievirus A16 reveals the emergence of a new clade in mainland China. Arch Virol.

[13] Hassel C, Mirand A, Farkas A, Diedrich S, Huemer HP, Peigue-Lafeuille H (2017). Phylogeography of coxsackievirus A16 reveals global transmission pathways and recent emergence and spread of a recombinant genogroup. J Virol.

[14] Wang J, Teng Z, Chu W, Fang F, Cui X, Guo X (2018). The emergence and spread of one Coxsackievirus A16 Genogroup D novel recombinant strain that caused a clustering HFMD outbreak in Shanghai, China, 2016. Emerg Microbes Infect.

[15] Zhu RN, Qian Y, Deng J, Xing JF, Zhao LQ, Wang F (2007). Study on the association of hand, foot and mouth disease and enterovirus 71/CA16 among children in Beijing, 2007. Zhonghua Liu Xing Bing Xue Za Zhi.

[16] Song Q, Huang H, Deng J, Zhao L, Deng L, Sun Y (2015). Analysis on the change of genotype of enteroviruses associated hand, foot and mouth disease in Beijing during 2013 to 2014. Zhonghua Er Ke Za Zhi.

[17] Yu FY, Zhu RN, Deng J, Song QW, Jia LP, Liu LY (2018). Pathogen spectrum in enteroviral infections among children in Beijing from 2010 to 2016. Zhonghua Er Ke Za Zhi.

[18] Tamura K, Stecher G, Peterson D, Filipski A, Kumar S (2013). MEGA6: molecular evolutionary genetics analysis version 6.0. Mol Biol Evol.

[19] Rambaut A, Lam TT, Max Carvalho L, Pybus OG (2016). Exploring the temporal structure of heterochronous sequences using TempEst (formerly Path-O-Gen). Virus Evol..

[20] Suchard MA, Lemey P, Baele G, Ayres DL, Drummond AJ, Rambaut A (2018). Bayesian phylogenetic and phylodynamic data integration using BEAST 1.10. Virus Evol..

[21] Darriba D, Taboada GL, Doallo R, Posada D (2012). jModelTest 2: more models, new heuristics and parallel computing. Nat Methods.

[22] Murrell B, Wertheim JO, Moola S, Weighill T, Scheffler K, Kosakovsky Pond SL (2012). Detecting individual sites subject to episodic diversifying selection. PLoS Genet.

[23] Kosakovsky Pond SL, Frost SDW (2005). Not so different after all: a comparison of methods for detecting amino acid sites under selection. Mol Biol Evol.

[24] Murrell B, Moola S, Mabona A, Weighill T, Sheward D, Kosakovsky Pond SL (2013). FUBAR: a fast, unconstrained bayesian approximation for inferring selection. Mol Biol Evol.

[25] Lu J, Zeng H, Zheng H, Yi L, Guo X, Liu L (2014). Hand, foot and mouth disease in Guangdong, China, in 2013: new trends in the continuing epidemic. Clin Microbiol Infect.

[26] Hu YQ, Xie GC, Li DD, Pang LL, Xie J, Wang P (2015). Prevalence of coxsackievirus A6 and enterovirus 71 in hand, foot and mouth disease in Nanjing, China in 2013. Pediatr Infect Dis J.

[27] Shi J, Huang X, Liu Q, Huang Z (2013). Identification of conserved neutralizing linear epitopes within the VP1 protein of coxsackievirus A16. Vaccine.

[28] He S, Chen M, Wu W, Yan Q, Zhuo Z, Su X (2018). An emerging and expanding clade accounts for the persistent outbreak of coxsackievirus A6-associated hand, foot, and mouth disease in China since 2013. Virology.

[29] Han Z, Song Y, Xiao J, Jiang L, Huang W, Wei H (2020). Genomic epidemiology of coxsackievirus A16 in mainland of China, 2000–18. Virus Evol..

[30] Puenpa J, Wanlapakorn N, Vongpunsawad S, Poovorawan Y (2019). The history of enterovirus A71 outbreaks and molecular epidemiology in the Asia-Pacific region. J Biomed Sci.

[31] Meng XD, Tong Y, Wei ZN, Wang L, Mai JY, Wu Y (2020). Epidemical and etiological study on hand, foot and mouth disease following EV-A71 vaccination in Xiangyang. China Sci Rep.

[32] Qiu J, Yan H, Cheng N, Lu X, Hu X, Liang L (2019). The clinical and epidemiological study of children with hand, foot, and mouth disease in Hunan, China from 2013 to 2017. Sci Rep.

[33] Koh WM, Bogich T, Siegel K, Jin J, Chong EY, Tan CY (2016). The epidemiology of hand, foot and mouth disease in Asia: a systematic review and analysis. Pediatr Infect Dis J.

[34] Chen X, Tan X, Li J, Jin Y, Gong L, Hong M (2013). Molecular epidemiology of coxsackievirus A16: intratype and prevalent intertype recombination identified. PLoS ONE.

[35] Chan YF, Wee KL, Chiam CW, Khor CS, Chan SY, Amalina WM (2012). Comparative genetic analysis of *VP4*, *VP1* and *3D* gene regions of enterovirus 71 and coxsackievirus A16 circulating in Malaysia between 1997–2008. Trop Biomed.

[36] Noisumdaeng P, Korkusol A, Prasertsopon J, Sangsiriwut K, Chokephaibulkit K, Mungaomklang A (2019). Longitudinal study on enterovirus A71 and coxsackievirus A16 genotype/subgenotype replacements in hand, foot and mouth disease patients in Thailand, 2000–2017. Int J Infect Dis.

[37] Zhao K, Han X, Wang G, Hu W, Zhang W, Yu XF (2011). Circulating coxsackievirus A16 identified as recombinant type A human enterovirus. China Emerg Infect Dis.

[38] Zhang Y, Wang D, Yan D, Zhu S, Liu J, Wang H (2010). Molecular evidence of persistent epidemic and evolution of subgenotype B1 coxsackievirus A16-associated hand, foot, and mouth disease in China. J Clin Microbiol.

[39] Zhang M, Zhao Y, Zhang H, Lin K, Liu H, Zhang J (2019). Molecular characterization of Coxsackievirus A16 strains isolated from children with severe hand, foot, and mouth disease in Yunnan, Southwest China, during 2009–2015. J Med Virol.

[40] Sun Z, Zhang G, Guo P, Liu J, Gao Q, Xu X (2017). Epidemiological characterizations, pathogen spectrum and molecular characteristics of coxsackievirus A16 from patients with HFMD in Yantai, Shandong, China between 2011 and 2015. Hum Vaccin Immunother.

[41] Zhao G, Zhang X, Wang C, Wang G, Li F (2016). Characterization of VP1 sequence of coxsackievirus A16 isolates by Bayesian evolutionary method. Virol J.

[42] Palani S, Nagarajan M, Biswas AK, Maile A, Paluru V (2016). B1c genetic subtype of coxsackievirus A16 associated with hand, foot and mouth disease in Andaman Islands, India. Trans R Soc Trop Med Hyg.

[43] Bentley K, Evans DJ (2018). Mechanisms and consequences of positive-strand RNA virus recombination. J Gen Virol.

[44] Lukashev AN, Vakulenko YA (2017). Molecular evolution of types in non-polio enteroviruses. J Gen Virol.

[45] Racaniello VR. Picornaviridae: the viruses and their replication. In: Knipe DM, Howley PM, editors. Fields virology. 6th ed. Philadelphia: Lippincott Williams & Wilkins; 2013. p. 453–89.

[46] Ren J, Wang X, Hu Z, Gao Q, Sun Y, Li X (2013). Picornavirus uncoating intermediate captured in atomic detail. Nat Commun.

[47] Ma Z, Zha J (2016). Characterization of VP1 gene of coxsackievirus A16 prevalent among hand foot mouth disease suffered children in Taizhou, P. R. China, between 2010 and 2013. J Med Virol.

[48] Chen X, Zhang Q, Li J, Cao W, Zhang JX, Zhang L (2010). Analysis of recombination and natural selection in human enterovirus 71. Virology.

