# Supplementary figures and images for: Molecular epidemiology of coxsackievirus A16 circulating in children in Beijing, China from 2010 to 2019
Source: World J Pediatr. 2021 Aug 28;17(5):508–16. doi: 10.1007/s12519-021-00451-y (PMC8523403; doi:10.1007/s12519-021-00451-y)

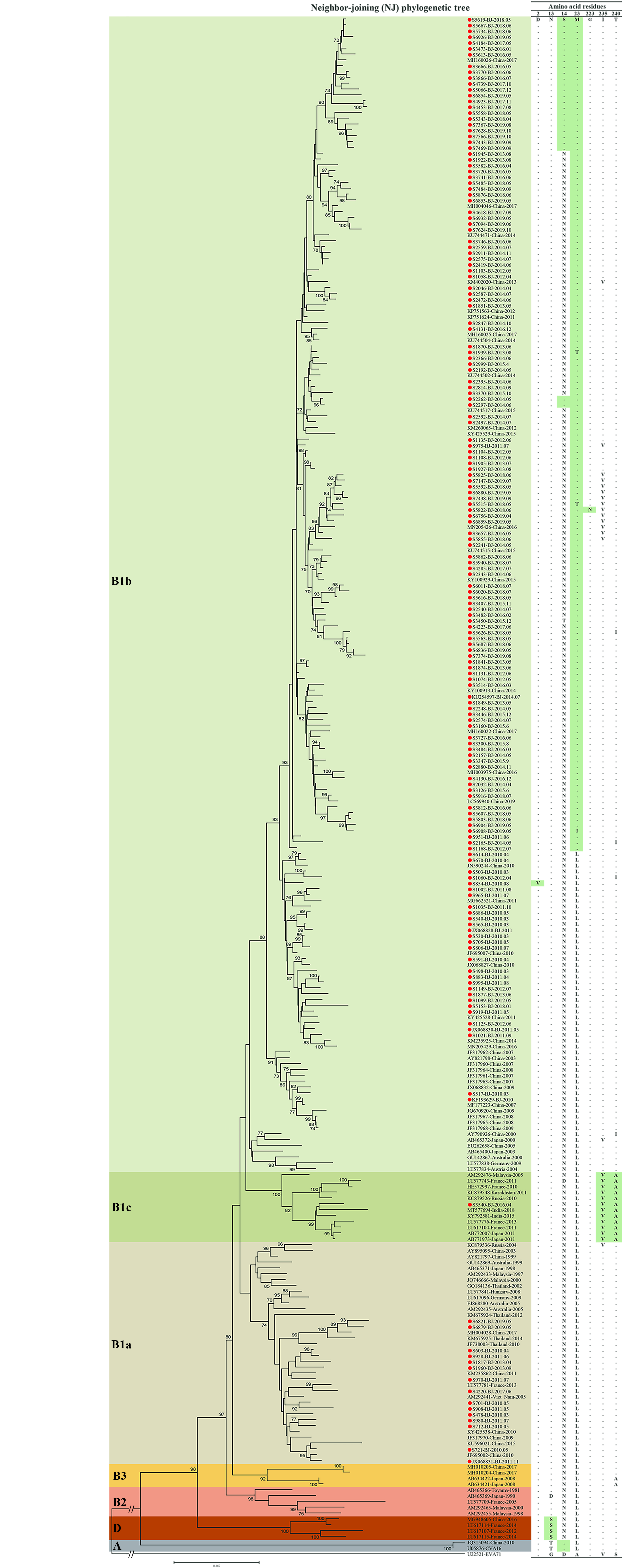

Supplement: Supplementary file 1 — Supplementary Fig. 1 Neighbor-joining (NJ) phylogenetic tree based on the nucleotide sequences of the CVA16 VP1 gene and alignment of the deduced amino acid residues of VP1 protein. Duplicate sequences obtained in this study were deleted. The prototype enteroviruses A71 strain (BrCr) was used as an outgroup. The nucleotide substitution model was Kimura 2-parameter. A bootstrapping analysis was performed 1000 pseudo-replicated datasets. Only bootstrap support values ≥ 70% were shown. The strains collected in Beijing during 2010–2019 were marked with red circle. Deduced VP1 amino acid residues alignment was listed on the right. Mutations that are specific among genotypes/sub-genotypes and under positive selection pressure are highlighted with green. CVA16 coxsackievirus A16, VP viral protein (TIF 13940 kb) [file 12519_2021_451_MOESM1_ESM.tif]

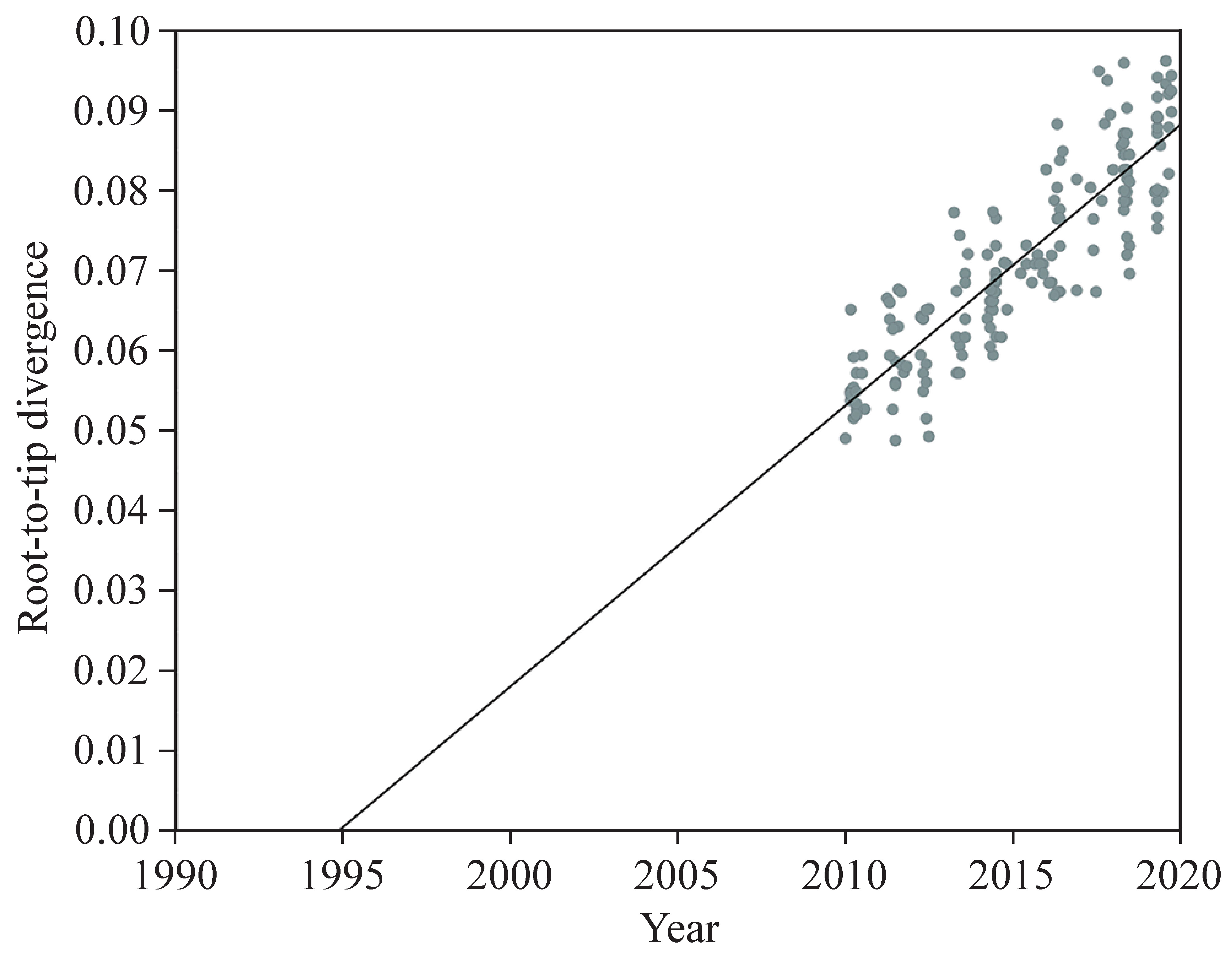

Supplement: Supplementary file 3 — Supplementary Fig. 3 Root-to-tip regression analysis for the temporal signal of the VP1 gene of CVA16 circulating in Beijing children during 2010-2019 calculated by TempEst. VP viral protein, CVA16 coxsackievirus A16 (TIF 670 kb) [file 12519_2021_451_MOESM3_ESM.tif]
